# Revisiting the fluid challenge and stroke volume optimisation after induction of general anaesthesia

**DOI:** 10.1016/j.aicoj.2026.100093

**Published:** 2026-05-22

**Authors:** Terry O’Brien, Robert G. Hahn

**Affiliations:** Karolinska Institutet at Danderyds Hospital (KIDS), Stockholm, Sweden

**Keywords:** Fluid challenge, Crystalloid, Colloid fluid

## Abstract

**Background:**

To be clinically useful in goal-directed fluid therapy, a fluid challenge (FC) must achieve a large enough blood volume change (ΔBV) to materially change ventricular preload and stroke volume (ΔSV). The same % change in BV for all the subsequent FCs must also be achieved. We explored under what circumstances these prerequisites can be met by referring to data from a clinical trial where 111 patients underwent three successive intravenous FCs using crystalloid (Ringer´s lactate) or colloid (hydroxyethyl starch), involving a total of 9 mL/kg of fluid, just after induction of general anaesthesia.

**Results:**

The three FC using crystalloid fluid increased SV by 2.5% while the colloid increased the SV by 29.2% (medians, *P* < 0.001). Moreover, crystalloid expanded the BV by 20.8% while the corresponding expansion by colloid was 29.5% (*P* < 0.001). The cardiac response to increased BV was normalized by calculating the ΔSV%/ΔBV% ratio, which was much lower at 0.12 for the crystalloid fluid versus 0.98 for colloid (*P* < 0.001). The poor SV response can possibly be explained by the underlying changes in the mean circulatory filling pressure, which suggest crystalloid FCs was less effective in increasing the stressed blood volume (preload). On the other hand, the inferior BV expanding effect of crystalloid is likely due to the fast tissue redistribution pharmacokinetic profile of administered boluses. Finally, the cardiac response to the first FC and impact on oxygen delivery became falsely low in both groups due to a reflex redistribution of a substantial volume of interstitial fluid into the circulation after anaesthetic induction.

**Conclusion:**

Differences in effectiveness between crystalloid and colloid fluid when providing repeated FCs increase the risk of false negative indications of fluid non-responsiveness when crystalloid is used.

## Background

Fluid challenges (FCs) are given to assess fluid responsiveness and position on the Starling curve [1]. In major surgery FCs are the cornerstone of the initial “optimising” phase of stroke volume (SV) in goal-directed therapy (GDT) protocols [2,3]. FCs were originally performed with colloid boluses which are repeated until the SV failed to change by ≥10%. Over the last 10 years concerns about the safety of colloid fluids resulted in transition to the use of crystalloids for the FC procedure in surgery [4]. Colloids induce a long-term expansion of the blood volume (BV) while crystalloids show a prominent distribution phase which gives them a much shorter intravascular half-life. However, the consensus was that the change in fluid would not impact the effectiveness of GDT protocols [5,6]. The beneficial effect of GDT on postoperative complication rates has since then become less clear [3,[7], [8], [9]] which might be due to the adoption of enhanced recovery protocols, more restrictive fluid management and the use of less invasive surgery. However, the change to use crystalloid instead of colloid fluid might also have contributed to the loss of effectiveness of GDT.

For a FC to be clinically useful it is required that it achieves a large enough BV change to materially change the SV and that the same % change in BV is achieved for all the subsequent FCs. These requirements are important because the definition of fluid responsiveness is currently a fixed value, i.e., a change in SV of ≥10%. Our previous paper [10] used an in-silico model to show that the pharmacokinetic profiles of colloid and crystalloid FCs differ significantly in their impact on BV expansion (ΔBV). We also pointed out that measurements may be confused by the capillary in-flow of fluid that occurs in the first 20 min post anaesthetic induction in response to a fall in arterial pressure [11].

In the present study, we retrieved data on ΔSV and ΔBV from a clinical trial to compare how these variables respond to a series of FCs. The primary aim is to study the development of their responses to a sequence of colloid or crystalloid FCs of equal dose given immediately post anaesthesia in surgical patients. Secondary aims were to estimate the volume and impact of post anaesthesia capillary refill on the ΔBV. We introduce a new variable, SV%/ΔBV%, to specifically describe the heart´s ability to handle increased BV, which then helps distinguish the cardiac response from the confounding influence of pharmacokinetic differences between the infusion fluids. Additionally, the data allowed us to examine the usefulness of pre surgery crystalloid fluid loading.

## Materials and methods

### Patients

The study is a secondary analysis of 111 patients with suspected or established gastric, colonic or rectal cancer were recruited to participate in this open-labelled randomized clinical trial [12,13]. They were scheduled for laparoscopic or open gastrointestinal surgery under combined intravenous (i.v.) and inhalational general anaesthesia. Exclusion criteria were liver or renal dysfunction (liver enzymes >50% or serum creatinine >50% of normal), coagulation disturbances, obstructive pulmonary disease, atrial fibrillation and mental disorders.

### Anaesthesia

After an overnight fast, at 8 AM, general anaesthesia was initiated after giving 2–4 L of oxygen on an open facemask. The drugs used were i.v. lidocaine, fentanyl and propofol. The propofol dose was guided by a TCI protocol aiming at a plasma concentration of 3−4 μg/mL. Tracheal intubation was facilitated with cisatracurium 0.2 mg/kg. The patients were mechanically ventilated using a tidal volume of 8 mL/kg and positive end-expiratory pressure of 3 cm H_2_O. The ventilation rate was 12 breaths/min or adjusted to maintain end-tidal CO_2_ at 36–44 mmHg. The inspiratory-expiratory ratio was 1:2.

Depth of anaesthesia was monitored by a bispectral index sensor applied to the forehead. The signal was recorded on a BIS monitor Model A-2000TM (Aspect Medical System, Natick, MA) and the anaesthesia guided to reach a BIS value of between 40 and 60. The drugs used for maintaining anaesthesia was 1–2% end-tidal sevoflurane, continuous infusion of propofol (target plasma concentration, 2–3 μg/mL) and/or remifentanil (0.10–0.20 μg/kg/min) and cis-atracurium intermittently as needed. Body temperature was maintained at 35.5 °C or higher.

### Fluid programs

No fluid was infused during the induction of anaesthesia. Beginning 10 min after the tracheal intubation, each patient received three successive FCs to achieve a total fluid dose of 9 mL/kg according to one of the following programs.

1 FCs with colloid but preloading with 500 mL of Ringer over 2 h was given, beginning 3 h before the induction of anaesthesia.
2 Colloid fluid only.
3 Ringer only.

Each of the three FCs consisted of 3 mL/kg of fluid administered over fluid 7 min. A total of 9 mL/kg of fluid was given to all patients via an infusion pump (IEC 601–1; Abbott Laboratories, Chicago, IL). The flat recumbent body position was maintained, and surgery was not started until all three optimizations had been completed.

The colloid fluid used was 6% hydroxyethyl starch 130/0.4 (Voluven®; Fresenius Kabi Deutschland GmbH, Bad Homburg, Germany) or Ringer´s lactate (Fresenius).

### Measurements

One radial artery was catheterized before anaesthesia was induced with the aid of local anaesthesia and sedation by midazolam. The arterial line was connected to a FloTrac™ sensor from which data was sent to a Vigileo monitor (Software version 3.6; Edwards Lifesciences, Irvine, CA). The monitor reported the mean arterial pressure (MAP) and the arterial waveform pulse contour, which was used to calculate stroke volume (SV). Moreover, the central venous pressure (CVP) was measured via a catheter introduced via the internal jugular vein. The CVP was calibrated before the anaesthesia was induced. Zero corresponded to the level of 4th rib in the anterior axillary line. Data on pulse oximetry, electrocardiography and heart rate were saved digitally (Datex-Ohmeda, Hoevelaken, the Netherlands). The central haemodynamic variables were recorded before and after induction of anaesthesia, just before the first bolus infusion was initiated, and then 5 min after each one of the bolus infusions ended.

Arterial blood was withdrawn for measurement of the blood haemoglobin (Hb) concentration, the Hb oxygen saturation (SO_2_) and the oxygen tension (*p*O2) on EM Premier 3000 (Instrumentation Lab., Lexington, IL) at the same time as central haemodynamics was measured. Duplicate baseline samples yielded a coefficient of variation for Hb of 1.5%.

### Calculations and definitions

***Blood volume.*** The blood volume (BV) changes in response to the bolus infusions were calculated by multiplying the change in the Hb concentration with the baseline BV, which was estimated based on the height and weight of each patient as follows [14]:

BV (L, females) = 0.03308 weight (kg) + 0.3561 height^3^ (m) + 0.1833

BV (L, males) = 0.03219 weight (kg) + 0.3669 height^3^ (m) + 0.6041

The BV expansion in response to a fluid bolus was calculated from before (time 1) and after (time 2) the infusion according to the following equation [15]:

ΔBV_2−1_ = BV_1_ (Hb_1_/Hb_2_) – BV_1_

***Fluid responsiveness.*** The patient was a ***fluid responder*** when an FC raised SV by ≥10.0% and a ***non-responder*** when the increase was <10.0% [[1], [2], [3]]. The target in flow-guided optimization with FCs is to reach the top of the Frank-Starling curve limiting the volume administered through assessment of the SV response. However, in this study the three FCs were given without consideration of the SV response, thus all patients received a cumulative FC dose of 9 mL/kg.

***Guyton´s parameters.*** An analogue to the mean circulatory filling pressure (P_msa_) has been derived from measurements CO, MAP, and CVP, assuming a constant veno-arterial compliance of 24:1 [[16], [17], [18]]. The equations used to calculate P_msa_ and the associated “Guyton’s haemodynamic parameters” is explained in Supplementary File: Calculation of Guyton’s parameters.

***Capillary refill autotransfusion.*** Translocation of interstitial fluid into the plasma is initiated by the induction of anaesthesia. This fluid transfer during the FCs was taken as being equal to the difference between the estimated BV change for colloid fluid and the known acute blood volume expanding property of the starch solution used. Voluven was found to acutely expand the BV by 1.2 times the infused volume before surgery [19] while the expansion was 1.0 in volunteers not scheduled for surgery [20]. We used the factor 1.2 and the infused colloid volume (mean, 180 mL) was then expected to expand the BV by 180 mL *1.2 = 216 mL.

### Statistics

The haemodynamic variables and BV were reported as the median and 25th-75th percentile range. Data having a normal distribution are presented as mean ± standard deviation (SD). Incidence data were tested by contingency table analysis. The serial changes in the key variables (BV, SV, and ΔSV%/ΔBV%) with respect to the choice of fluid was studied by repeated-measures ANOVA. The differences between the three groups in response to each FC were further studied by the Kruskal–Wallis test after which *post hoc* comparisons between the groups were made using the pairwise test implemented in SPSS version 30.0.0 for Mac (IBM Corp., Armonk, NY). As the two colloid groups showed very similar behaviour, selected comparisons between the pooled colloid groups and the Ringer-only group were made using the Mann–Whitney U test and two-way ANOVA. The 95% “best” estimate and the 95% confidence interval (CI) for the difference these two groups were given using the Hodges-Lehmann method. Power analysis was not performed due to the secondary nature of pour analysis. Significance was defined as *P* < 0.05.

## Results

There were no statistically significant differences between the three groups with respect to age, body weight, sex distribution, and baseline SV and BV (Table 1).

**Table 1:** Patient details and BV & SV baseline values.

Patient Details	Ringer preload + Colloid	Colloid	Ringer’s
N	30	56	25
Age (years)	57.7 ± 13.4	55.7 ± 12.3	61.4 ± 9.3
Body weight (kg)	58.5 ± 8.7	60.4 ± 8.1	56.7 ± 8.8
Males (%)	57	70	72
Baseline SV & BV			
SV (mL)	78.7 ± 17.5	84.5 ± 28.8	86.4 ± 17.8
BV (L)	4.02 ± 0.84	4.39 ± 0.74	4.13 ± 0.80

SV, stroke volume; BV, blood volume.

No between-group differences found by one way ANOVA.

### Anaesthesia effects

The induction of general anaesthesia had a marked effect on the haemodynamics.

SV decreased from 82 (67–97) to 49 (40–61) mL/beat, MAP from 104 (97–112) to 79 (73–86) mmHg, and P_msa_ from 14.2 (12.4–15.4) to 10.7 (9.3–12.2) mmHg (all changes, *P* < 0.001). By contrast, the CVP was unchanged at 5 (3–6) mmHg. None of these changes differed significantly between the groups. The haemodynamic patterns are shown in Fig. S2A and S2B of Supplementary File 1.

The BV increased from 4.33 (3.51–4.87) to 5.04 (4.12–5.66) L (*P* < 0.001) despite that no fluid was infused. One patient received i.v. ephedrine 10 mg when the mean arterial pressure (MAP) fell to <65 mmHg. No other vasopressor was administered during the study.

### SV responses

**Fig. 1:**
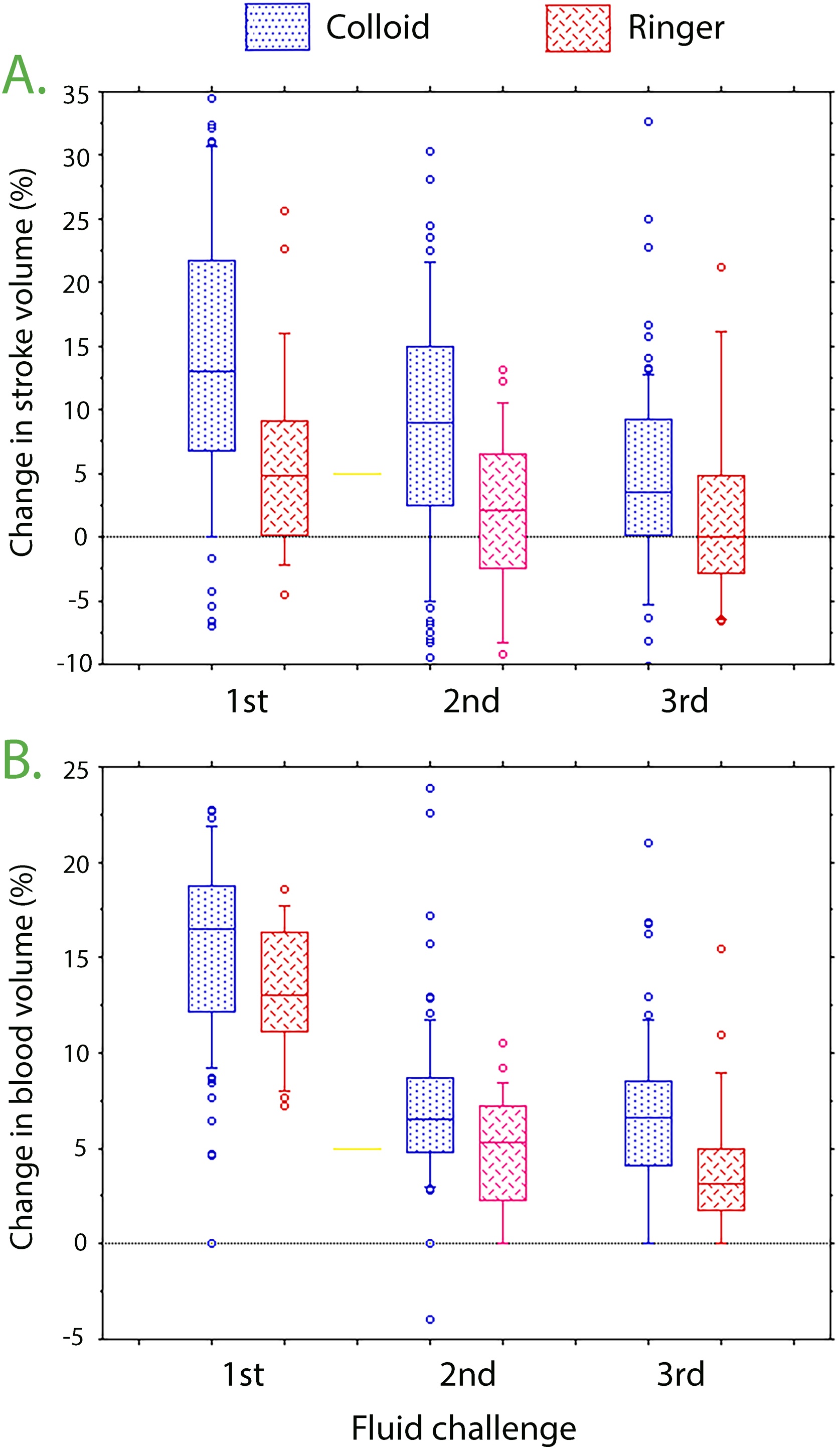
Box-plots showing the changes in (A) stroke volume, SV, and (B) blood volume, BV, in response to 3 consecutive bolus infusions of 3 mL/kg of colloid* (Voluven) and crystalloid fluid (Ringer) over 7 min. Note SV & BV responses are pooled from both Ringer + colloid and Colloid FC subjects.

The three FCs increased the SV (*P* < 0.001, F-value 12.5) and the SV responses differed significantly between the fluids (*P* < 0.013, F-test 7.1) with a non-significant interaction effect (P = 0.081, F-value 2.1; repeated-measures ANOVA). Specifically, the SV response was consistently weaker when Ringer was infused as compared to when a colloid was administered (Table 2, top). A difference was noted early on – the SV increase during FC1 was only 35% of the SV response to the colloids (ratio based on % median values), the second bolus averaged 23%, while no SV change at all occurred in response the third Ringer bolus (Fig. 1A).

**Table 2:** Changes in stroke volume and blood volume following three sequential fluid challenges (FCs).

Variable change	Ringer’s Inf. + Colloid (1)	Colloid (2)	Ringer’s (3)	P-value
STROKE VOLUME (mL/beat, %)				
1st	6 (13.1%)	6 (13.3%)	3 (4.8%)	0.001; 3 < 1, 2
2nd	4 (7.7%)	5.5 (9.2%)	1 (2.1%)	0.001; 3 < 1, 2
3rd	3.5 (5.5%)	2 (2.8%)	0 (0%)	0.14
All 3	14.5 (29.9%)	14.0 (28.9%)	1.5 (2.5%)	0.001; 3 < 1,2
BLOOD VOLUME (mL, %)				
1st	629 (14.2%)	718 (17.4%)	551 (13.1%)	0.012; 3 < 2
2nd	245 (6.2%)	302 (6.9%)	219 (5.4%)	0.005; 3 < 2
3rd	266 (7.0%)	263 (6.2%)	114 (3.2%)	0.001; 3 < 1, 2
All 3	1,127 (29.0%)	1,270 (29.8%)	862 (20.8%)	0.001; 3 < 1, 2
STROKE VOLUME (%) / BLOOD VOLUME (%)				
1st	0.86 (0.63–1.41)	0.74 (0.36–1.46)	0.38 (0.00–0.76)	0.013; 3 < 1, 2
2nd	0.92 (0.25–2.53)	1.14 (0.39–1.93)	0.27 (−0.50–1.29)	0.29
3rd	0.59 (0.00–1.68)	0.34 (−0.17–1.53)	0.00 (−0.60–0.88)	0.30
All 3	1.11 (0.60–1.83)	0.93 (0.40–1.55)	0.12 (−0.10–0.66)	0.004; 3 < 1, 2

Table is modified from Hahn, He & Li [12]. Median values are shown and Kruskal–Wallis test with post hoc analysis using the Bonferroni correction for multiple tests was used. The Stroke volume / Blood volume (ΔSV/ΔBV) ratio is reported as median and 25th-75th percentile range.

The entire series of 3 FCs with Ringer increased the SV by only 10% as much as the colloid infusions. The best estimate and 95% confidence interval for the difference in total SV response between the colloid groups and the Ringer group was 11 (7–15) mL/beat or 21 (12–31%) (both *P* < 0.001 with Hodges-Lehman test).

### BV responses

Overall, the BV increased in response to the three bolus infusions (*P* < 0.001). However, the magnitude of the responses differed significantly between the fluids (*P* < 0.001) and they were clearly weaker for Ringer than for colloid (Table 2, middle). For example, the BV change to FC1 and FC2 in the Ringer group was 79% and 82% of the responses in the colloid groups, and the ratio decreased to 48% for FC3. The BV responses to FC2 and FC3 were virtually identical in the colloid groups (6.5% and 6.6%, respectively) while it decreased by a further 40% in the Ringer group (from 5.4% to 3.2%; Fig. 1B).

The estimated 95% confidence interval for the difference in total BV response between the colloid groups and Ringer was 373 (223–520) mL or 8 (5–11)% (both *P* < 0.001).

### ΔSV% / ΔBV% ratio

**Fig. 2:**
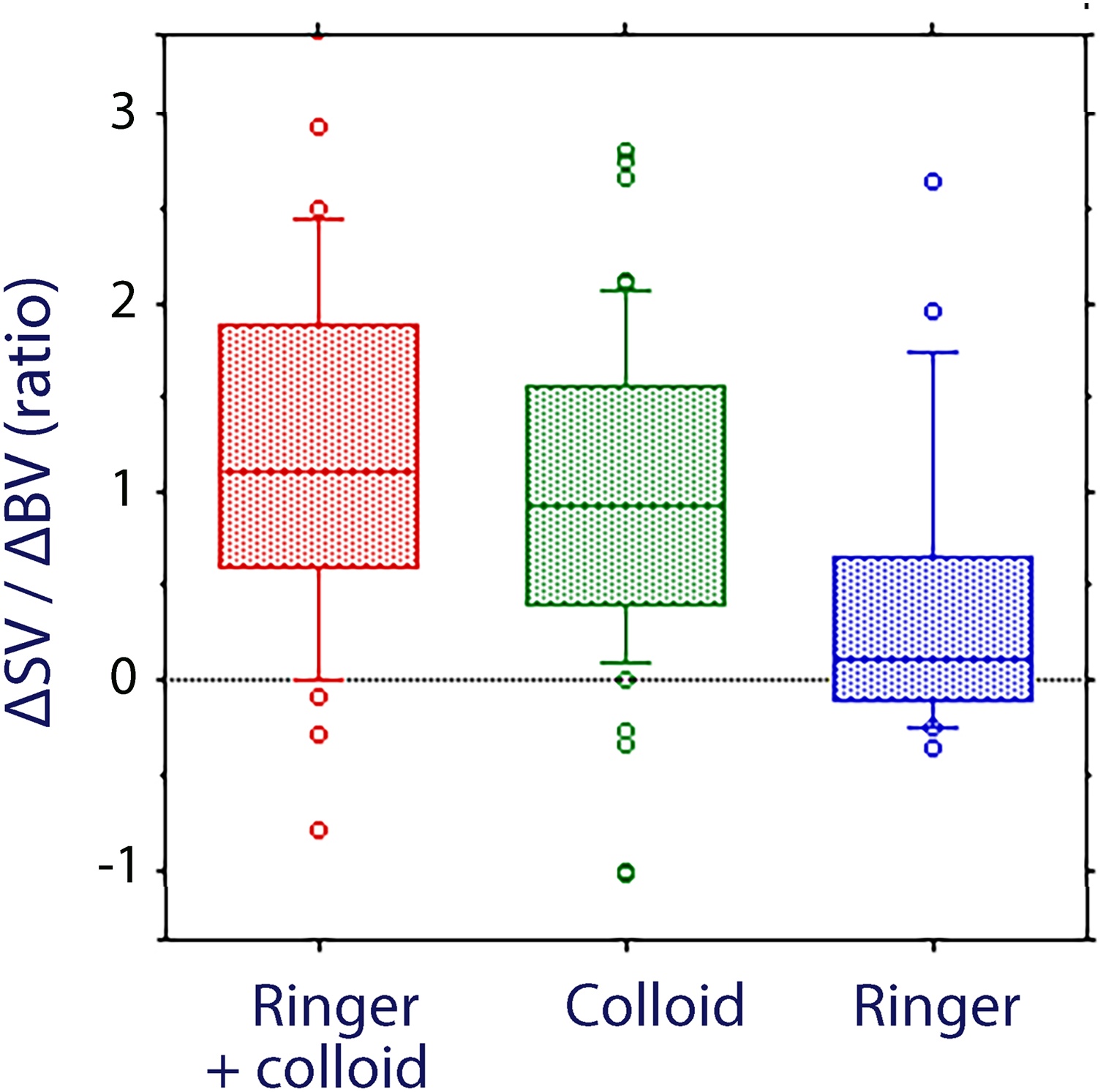
The total ΔSV%/ΔBV% ratio for 3 consecutive bolus infusions of 3 mL/kg of fluid according to 3 different programs.

The ratio of the FC-induced changes in SV and BV was used to express how responsive the heart is to increase SV following an increase in BV, which is used here as a surrogate for preload. This ΔSV%/ΔBV% ratio varied significantly in response to the fluid boluses (*P* < 0.04, F-value 3.8) and also between the fluids (*P* < 0.027, F-value 3.8; interaction effect P=0.51). Specifically, this cardiac effectiveness was only half as good for Ringers as for the colloids during the first FC, and the difference then decreased progressively (Table 2, bottom, and Fig. S1 of Supplementary File 1). By the third FC the median ΔSV%/ΔBV% ratio was 0, versus 0.59 and 0.34 for colloid FCs. When summarizing all the FCs the effectiveness of Ringer´s was only 10% of the colloid fluids (Fig. 2). Looking closer at this data shows that the mean fluid effectiveness value for colloid fluid responders was 2.0× vs 1.2× for Ringer and for fluid nonresponders 0.1× and −0.1×, respectively.

The estimated 95% confidence interval for the total difference in ΔSV%/ΔBV% ratio between the pooled colloid groups and Ringer was 0.67 (0.30–1.03, *P* < 0.008). This difference was consistent throughout the series of FCs although the range became wider it was 0.47 (0.20–0.78) for FC1, 0.84 (0.16–1.52) for FC1, and 0.57 (−0.02)–1.15) for FC3.

### Cardiac output, heart rate and arterial pressure responses

CO, MAP and oxygen delivery (DO_2_) were greatly affected by the induction of general anaesthesia but modestly impacted by the FCs (see Fig. S2A of Supplementary File 1). Despite significant increases in SV with colloid FCs, reflex decreases in HR meant the CO only increased across the FC series by a median of +3.1 [(−8.6)–14.7]%. HR decreased also in the Ringer group, resulting in that the lower SV increase (compared to the colloids) was reversed to a median CO reduction of −9.4 [(−14.1)–2.8]% after the 3 FCs (Mann–Whitney’s test *P* < 0.002).

In the colloid group median MAP fell by −2.7 [(−9.7)–4.1]% while the decrease was −11.9 [(−16.7)–3.1)]% in the Ringer-only group.

### Starling curve analysis

**Fig. 3:**
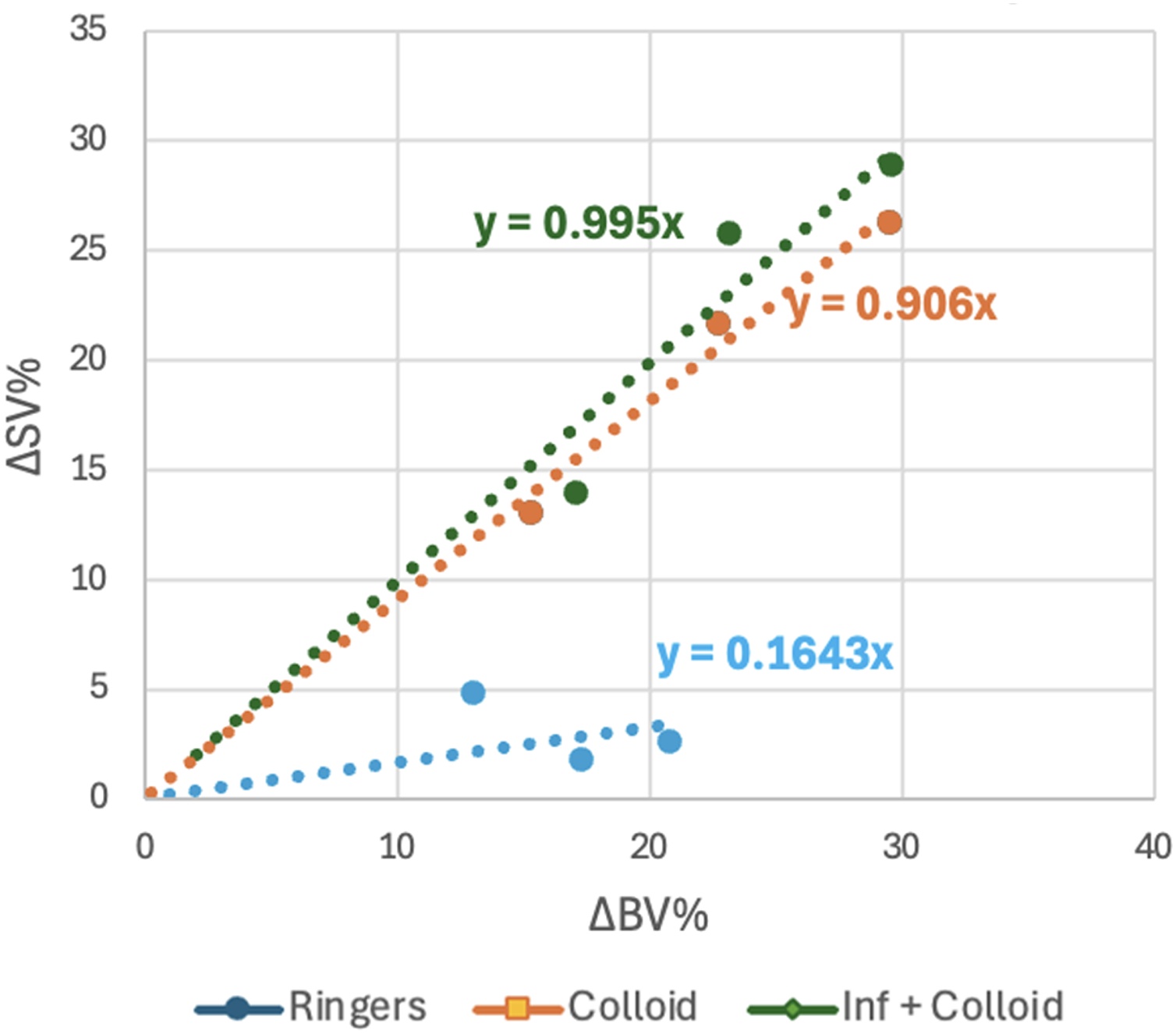
Starling curve analysis showing the cumulative median change in BV% and SV% for each of the 3 fluid interventions. The linear trendline data has been forced to 0 and the slope of the line indicated for each fluid. The graph shows that the pharmacokinetic property of Ringer fluid attenuates the BV expansion seen after administration of equivalent (9 ml/kg) volumes compared to colloid. This results in a BV expansion only 2 thirds of that observed with colloids. Despite the different ΔBV% expansion seen between the fluids, the slope of the curves should be similar if the ΔBV% equivalently impacted preload and cardiac contractility.

Fig. 3 combines the data shown in Table 2 and shows the cumulative median ΔSV% following each FC as a function of the cumulative median BV% changes for all three groups. This Starling curve-type analysis visually highlights differences between both the BV and the SV responses to colloid and Ringer FCs. The first point of both colloid curves begins to the right of the Ringer curve, and this reflects the difference in BV expansion strength during the first FC. The distance between each point projected on the x-axis becomes shorter with the Ringer FC, and this demonstrates the falling of the ΔBV change for sequential Ringers FCs.

### Mean circulatory filling pressure (P_msa_) and CVP

**Fig. 4:**
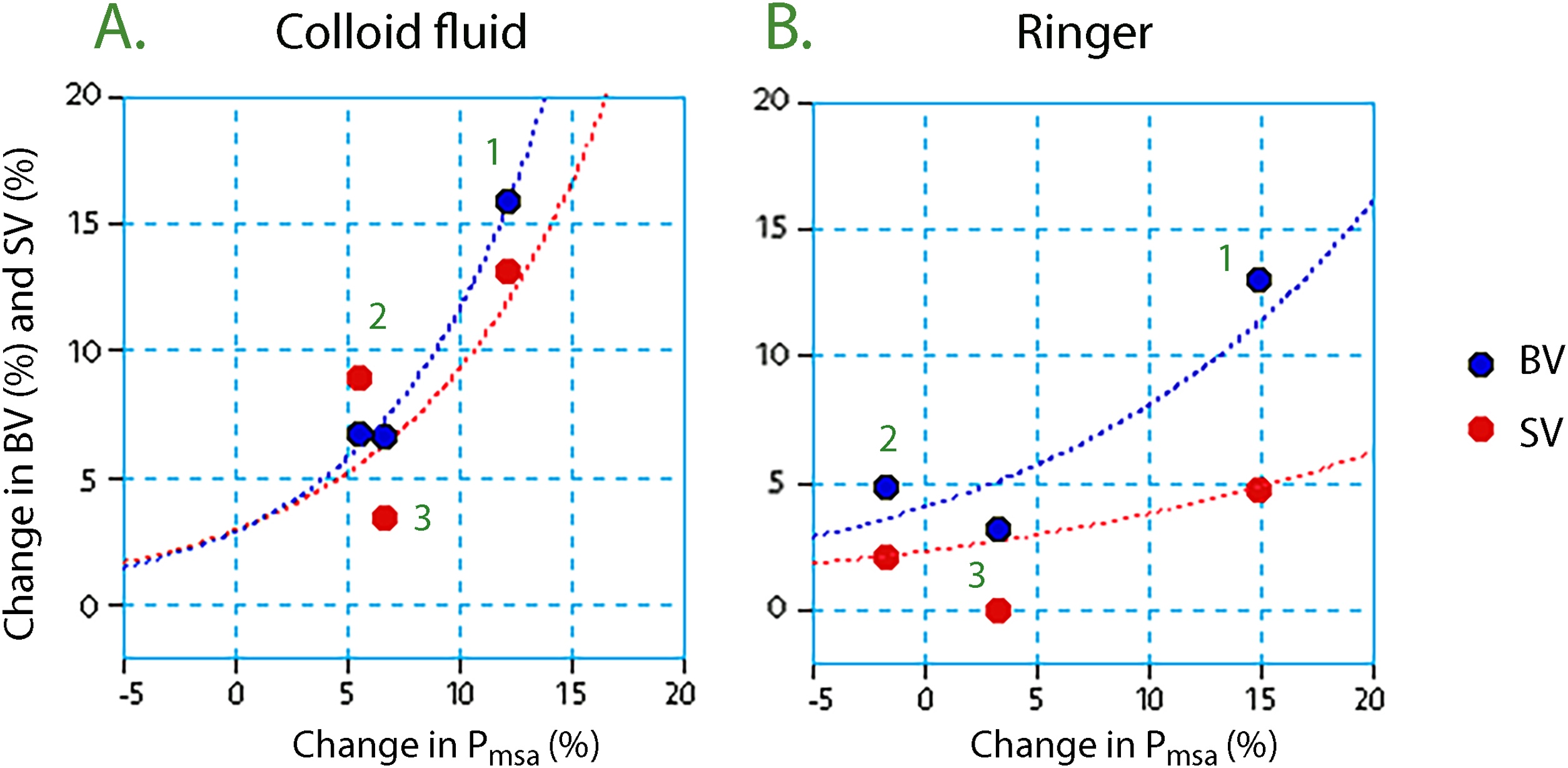
Relationship between FC-induced changes in mean circulatory filling pressure (P_msa_) and the corresponding changes in SV and BV. Median values, each point represents measurements performed at the end of one FC. The order of the measurements are marked with a number from 1 to 3.

The changes in P_msa_ at the end of each FC did not differ between the groups (P = 0.13) but the SV and BV responses to changes in P_msa_ were greater for colloid than for the crystalloid fluid (Fig. 4**)**. The median ratio ΔSV(%)/ΔP_msa_(%) for all 3 FCs was 7.2 (2.0–10.5) for the colloids but only 0.9 ((−0.8)–4.4) for the Ringer (difference 5.2 (2.4–7.6; *P* < 0.002)). Similarly, the ΔBV(%)/ΔP_msa_(%) for all 3 FCs was 2.7 (1.8–3.3) for the colloids and 1.5 (0.7–2.4) for the Ringer (difference 1.8 (0.3–1.7; *P* < 0.010).

Focusing attention on the second and third fluid challenges i.e. FCs administered after most of the endogenous capillary refill has occurred, give a cleaner picture of the impact of Ringer FCs on ΔBV% and its relationship with changes in P_msa_ and SV and suggest that for Ringer there is a lower, than colloid, impact on P_msa_ for an equivalent BV% change.

The CVP after induction and intubation before the first FC was 6.2 (4.0–7.8) mmHg for the colloid FCs and 6.8 (5.8–8.3) mmHg for Ringer FCs. The average CVP rose by approximately 10% for each FC with colloid. For the first Ringer FC, which was augmented by the capillary refill volume, the CVP increased (4.9–6.4). The CVP did not materially increase in response to Ringer administration across the next 2 FCs. The final CVP was up 35% at 8.3 (7.0–9.0) mmHg for the colloid FCs and was lower for Ringer FCs 6.8 (5.0–9.0) mmHg (*P* < 0.01).

In combination, the P_msa_ and CVP data suggest that most of the infused Ringer volume boluses (6 mL/kg) has ended up as “unstressed” volume and did not function as well as colloid to increase preload.

The time-course of the P_msa_ and other “Guyton variables” are illustrated in Fig. S2 in Supplementary File 1.

### Fluid responsiveness and SVV

Fluid responsiveness (SV increase *≥*10% resulting from a single FC) was twice as common when a colloid was used as compared to the Ringer alone group (43% vs. 18%, chi-square *P* < 0.002). The occurrence was 67%, 38%, and 23% for the preload + colloid group; 63%, 50% and 26% for the colloid-only group, and 24%, 17% and 13% for the Ringer-only group.

A higher stroke volume variation (SVV) was consistently needed for a subsequent FC to show fluid responsiveness in the Ringer-only group as compared to the two colloid groups (*P* < 0.03, 0.006, and 0.001 for the three FCs, respectively; two-way ANOVA). Overall, SVV averaged 2.7 percentages higher for non-responders and 5.6 higher for responders. The difference was greatest for the third FC. Here, the SVV was 9.5 ± 3.4% and 11.5 ± 3.2% for non-responders and responders in the colloid groups while the corresponding values were 14.0 ± 7.0% and 17.6 ± 3.1% for the Ringer-only group.

### Capillary refill autotransfusion

**Fig. 5:**
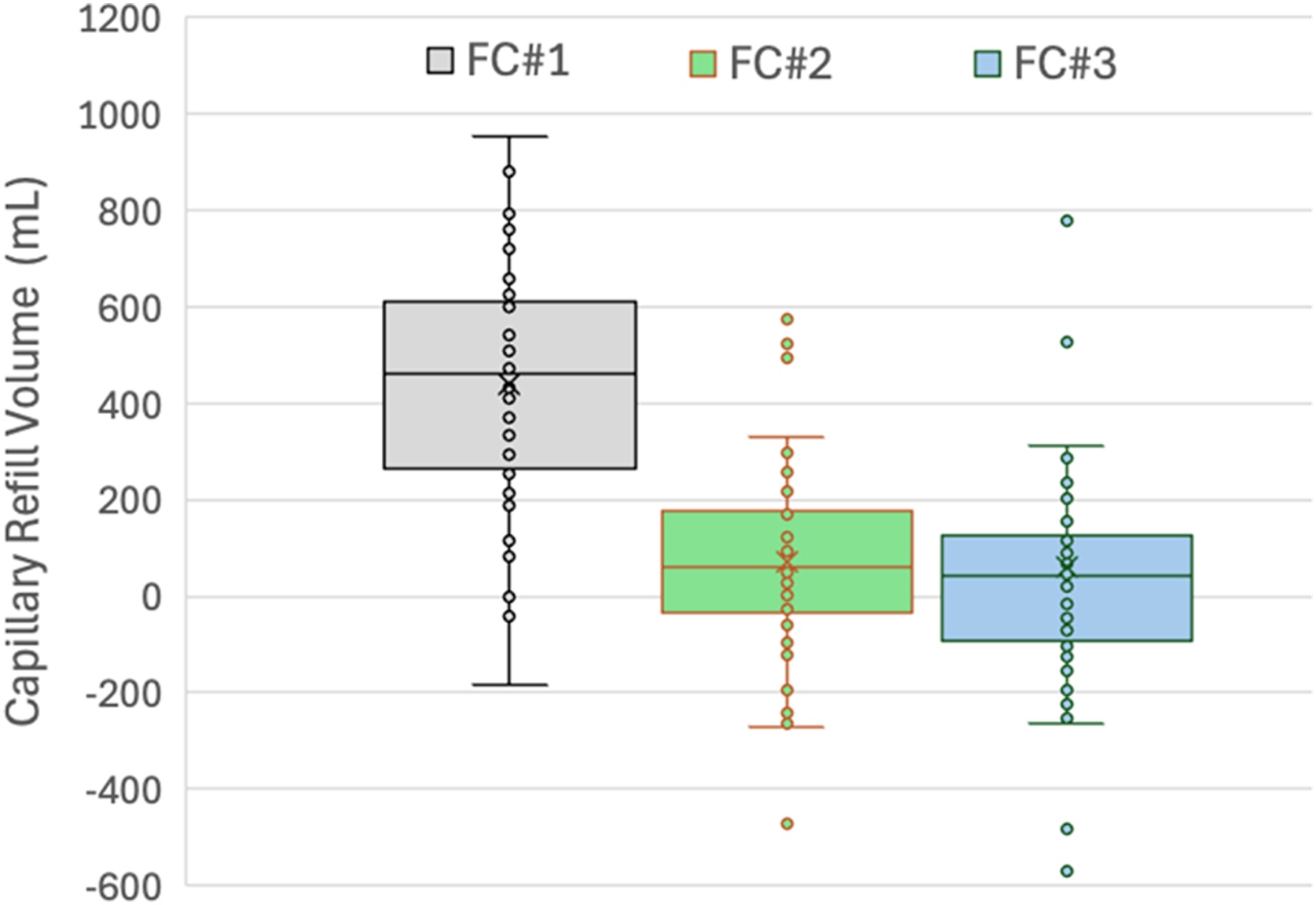
Estimated capillary refill volume for three consecutive fluid boluses of colloid fluid. The Box and Whisker chart shows: the mean, median, IQR, maximum/minimum and any outlier values.

An exploratory estimation of the autotransfusion of fluid that occurred in response to the induction of anaesthesia was made. The autotransfusion amounted to 444 (312–623) mL in the colloid groups during the first FC, which is greater than the infused volume. There was virtually no difference between the two colloid groups regarding the magnitude of this autotransfusion. Thereafter, capillary refill had a negligible impact on ΔBV measured for the subsequent FCs (Fig. 5).

## Discussion

### Main findings

The study demonstrated that using a crystalloid fluid for FC is less effective than using a colloid. First, the BV expansion following an FC with Ringer was less pronounced compared to a colloid fluid. The difference increased with successive FCs (“attenuation”) which did not occur with the colloid fluid. Moreover, the fluid-dependent differences were even more pronounced for SV and, therefore, the ΔSV%/ΔBV% ratio was greater in the colloid groups than in the Ringer group. This discrepancy places the two fluids on different slopes of a Starling curve (Fig. 3).

Further evidence of fundamental differences between colloid fluid and Ringer regarding their effectiveness of increasing cardiac preload comes from the data of P_msa_, which perhaps better reflects the “stressed” BV than the calculation of BV changes based on haemodilution. Calculation of the ΔBV(%)/ΔP_msa_(%) ratio supports the contention that a smaller fraction of the Ringer FC ended up in the “stressed” BV, from where it could hardly increase preload.

The potential for cardiac interstitial oedema to be a contributing factor to the poorer SV response for an equivalent BV change seen with Ringer is discussed.

Hence, several fluid-related factors probably contribute to the marked difference in fluid responsiveness seen between the colloid groups (43%) and the Ringer group (18%). These include “attenuation” of the BV response, weaker SV response to BV expansion, and a smaller fraction of the ΔBV that increases P_msa_.

Finally, our data support that a reflex autotransfusion greatly affects the interpretation of key hemodynamic and oxygen delivery measurements associated with FCs performed after anaesthesia induction.

### Background

Previous studies indicate that the FC dose, rate and timing of the SV assessment are important variables. Aya et al. [21] determined what is the minimal volume required to perform an effective fluid challenge where crystalloid fluid (1, 2, 3, and 4 mL/kg) was administered as a single FC bolus in a 4-arm study. They found an increase in the incidence of fluid responsiveness as the volume infused increased (20, 35, 45, and 65%). They concluded that 4 mL/kg of crystalloid should be used for a FC to adequately increase preload. Schmidt et al. [22] found that infusing 250 mL of a crystalloid, or colloid fluid, over 5 min was more effective, causing a greater increase in SV than when delivered over 20 min. Messina et al. [23] similarly showed that the infusion time of a crystalloid bolus (4 mL/kg) affects the determination of fluid responsiveness. They also showed that the pharmacodynamic effect on SV fades rapidly within minutes of the end of the infusion – implying that the fluid responsiveness test is very time dependent, particularly when crystalloid is used for FCs.

These earlier studies made important inroads to our understanding of the FC methodology. They indicate that the dose, dose rate and timing of the assessment of fluid responsiveness can cause variation in the magnitude of the BV that alters the preload change. In these studies, the BV change at the time of the SV evaluation was not measured or estimated.

Quantitatively what is meant by an adequate/effective change in BV to test for fluid responsiveness has not ever been defined. We believe that our study is the first to measure BV changes after matched multiple FCs of the same volume of two different fluids immediately post induction. The BV data has allowed us to introduce the ΔSV%/ΔBV% parameter as a measure of how effectively the heart handles an increase of BV. This parameter could potentially be used to define a minimum % change in BV that ideally, we should be targeting for all FC boluses, when a SV response of ≥10% is used to define fluid responsiveness. We suggest this should be in the order of a ΔBV of 5–7% - approximately the change historically achieved by a fluid challenge series of 250 mL of colloid or following the first FC of crystalloid at a dose of 4 mL/kg.

### Capillary refill

This study confirms that there is a capillary refill process immediately after anaesthetic induction, although the estimated magnitude of the increase is based on data from previous work. Fluid kinetic models suggested this to be a reflex compensatory capillary fluid in-flow over the first 20 min post anaesthetic induction due to the fall in MAP [10,11,24].

Capillary inflow in humans under anaesthesia was noticed long ago by Bond in 1969 [25] who showed a significant correlation between the fall in systolic arterial pressure and fall in the Hb concentration, and again more recently [26] that this autotransfusion is reduced when blood pressure is restored to pre-induction levels. The relatively long duration (20 min) of the inflow suggests a lymphatic origin for the fluid [11]. It will be an issue irrespective of the fluid chosen for FCs. Capillary inflow adds a substantial volume to the circulation, probably up to 1 L overall and in this series was estimated at circa 450 mL running in at an average rate of 37 mL/min over the 12-min FC bolus and SV evaluation period. The second and third FC were much less affected by capillary refill and are more indicative of the BV impact of the infused fluids *per se.*

This capillary inflow has not been previously recognised as a potential confounder for FC evaluation. Its physiological impact seems to be to increase BV to limit the drop in P_msa_ after induction. Our exploratory analysis of the magnitude of this inflow and its differential hemodynamic vs dilutional impacts combine to make achieving any increase in oxygen delivery in a FC series conducted after anaesthetic induction more challenging than previously understood.

### The preconditions for the FC

The first requirement for conducting an effective FC is that the bolus infusion must cause a material change in BV. For the first FC with Ringer only 24% were deemed responders, which was 1/3 of the percentage reported when colloid was used. The BV expansion from FCs using Ringers at 3 mL/kg, was less than our suggested ΔBV% optimal target of 5%–7%. Inadequate dose by itself will result in a poor SV response compared to equivalent colloid doses and a higher false negative determination of fluid non responsiveness. This agrees with the recommendation on FC dose reported by Aya et al. [21].

The second requirement is that if a ≥10% change in SV is used to assess fluid responsiveness, this implies that the FC must achieve a constant change in BV (the denominator) for all FCs in a test series. However, our data confirms that Ringer FCs change the BV by decreasing amounts, which is a phenomenon that we call “attenuation”. The BV expansion achieved with colloid does not attenuate across a FC series.

Clearly for crystalloid FCs, larger doses than 3 mL/kg need to be used to minimise the chance of false negative determinations of fluid responsiveness. Aya et al. [21] have suggested that using a dose of 4 mL/kg over 5 min will provide an effective BV change for the first FC. However, they administered a single FC and did not explore if this held true for subsequent crystalloid FCs. We reported in our first paper [10] that if the 4 mL/kg recommended by Aya et al. [21] is used for the first FC, three FCs that each expand the BV to the same degree would require 1,225 mL (280 mL, 420 mL and 525 mL) in a patient weighing 70 kg. Escalating the crystalloid dose in this way is not current practice and would be clinically unattractive, but what we suggest must be done if a fixed threshold value for ΔSV of ≥10% is continued to be used to assess responsiveness. In the future, if continuous BV measurements, or estimates, could be available, the anticipated BV expansion of a crystalloid FC could be used to individualise the FC dose, derive the fluid effectiveness parameter (ΔSV%/ΔBV%) and dynamically adjust the definition of fluid response thus potentially taking into account FC bolus attenuation.

In contrast, colloid FCs have a BV expansion impact greater than the infused volume. Historically a standard colloid FC dose was at least 200–250 mL [27]. We estimate this dose would have expanded the BV of an 80 kg subject (5 L) by 5.2%–6.5%. In our series the colloid FC BV expansion impact of 3 mL/kg colloid FCs was always >6%, i.e., large enough to have avoided the risk of a false negative determination of a non-responsive state with all 3 FCs. This means that the same volume of colloid can be used for each FC and as distribution losses are small, the timing of the post FC SV assessment will be less critical.

Colloid looks to have been better matched than crystalloid for assessing fluid responsiveness, improving Starling curve position and handling the SV optimisation phase of a GDT protocol. Recognising the need to control: the fluid choice, dose/dose rate, timing of FCs and the SV evaluation were less obvious when colloid was being used.

### Starling curve and fluid effectiveness (ΔSV%/ΔBV%)

Fig. 2 and the bottom of Table 2 show analyses of the relationship between the changes in SV and BV obtained by our new parameter, ΔSV%/ΔBV%. This parameter can be used to define the % change in BV that we should be targeting for a FC bolus series which, if the current FC method is used, we estimate to be in the order of +5% to +7%. A FC achieving a 10% change in SV is therefore redefined as achieving an effectiveness ΔSV%/ΔBV% ratio of >1.3–2.0. Physiologically, this looks to be a reasonable expectation as it would increase preload sufficiently to adequately challenge the cardiac response and even increase DO_2_.

The Starling curve for Ringer is not just be a consequence of the low initial dose and subsequent lower “attenuating” BV changes seen for each FC. The ΔSV% response for a matched change in BV% also seems to be suppressed. Looking at colloid fluid responders there is an energetic SV response to BV increases for FCs, particular those administered after the capillary refill period, giving ΔSV%/ΔBV% ratios of approximately 2.0 × . For FCs using Ringer’s this ratio is approximately 50% lower than for FCs using colloid, implying a right shift in the Starling curve. How are we to interpret this right shift? The absence of an increase in P_msa_ or CVP for Ringer FCs 2 and 3 given after the capillary refill period, suggests that the slower build-up of BV is not translating into an equivalent (to colloid) preload increase. Therefore, the most likely reason for the poorer fluid effectiveness with Ringer could be due to that more of the ΔBV from Ringer becomes located outside the “stressed” BV.

There are only a few previous reports that have measured both the BV and SV changes following FC’s during surgery. Verheij et al. [28,29] studied the effect of saline and colloids during cardiac and major vascular surgery. They argued that the higher cardiac index (CI) seen with colloid loading was due to greater plasma volume expansion. However, reanalysing their data we see that following administration of 1,400 mL of colloid, the CI increased by +44% and the BV + 17% which gives a ΔSV%/ΔBV% ratio of 2.6. For saline, 1,800 mL increased CI by 3.3% and the BV 2.5% which yields a ΔSV%/ΔBV% ratio of 1.32. These ratios are similar to the ones we report.

Verheij et al. [28] also found that the COP (colloid oncotic pressure) after saline FCs fell from 16.8 to 15.4 mmHg but for colloid the COP rose up from 18.1 to 21.7 mmHg. A decrease in COP increases net fluid flow from capillaries and may enhance oedema formation.

The heart responds poorly to oedema formation. Laine & Allen [30] reported that CO fell by 40% when myocardial water content was increased by only 3.5%. We have no direct evidence of cardiac oedema occurring or dP/dT contractility measurements, so we cannot exclude or confirm this as an additional factor in the right shifted nature of the Ringer Starling curve.

Interestingly, non-responders for all fluids were more similar both having very low ΔSV%/ΔBV% ratios (colloid 0.1× and Ringers −0.1×), typically less than 10% of those seen with responders. Any unwarranted FC has the potential to quickly undermine any DO_2_ gains. Our study highlights that even the SV increases in responders do not necessarily translate into increases in CO, MAP or DO_2_ as in this series the HR often fell after the FCs. Additionally, for 20 min post-anaesthesia induction, arterial oxygen content was reduced through the ongoing parallel reflex distribution of interstitial fluids.

### Preloading with Ringer

Our analysis suggests that the pre-induction infusion of 500 mL of Ringer given before surgery was not useful. It did not help to limit the decreases in MAP, P_msa_ and the other hemodynamic parameter that occur during induction. There is no suggestion that it builds up a more robust pre-surgery BV. Post induction trends in ΔSV and ΔBV are not different to the colloid FC group. Our analysis of the DO_2_ given in Figure S2B of Supplementary File 1 suggests that the preloading had a small net dilutive impact on oxygen content by the time of the FCs. Overall, there appears to be no benefit from use of a pre surgery loading dose of crystalloid and certainly adds extra fluid and salt.

### Limitations

The SV(%)/BV(%) ratio did not differ significantly between all three study groups at all points in time, although the median values differed by several multiples (Table 2). This is probably due to heterogeneity or limited number of patients in the Ringer alone group. Differences were statistically significant with the ANOVA but the output weakened by occasional missing data.

Our suggestion that cardiac oedema might be an additional factor underlying the weak SV(%)/BV(%) response to volume loading with Ringer solution is speculative and needs to be confirmed by future studies that assess cardiac response coupled to blood volume and ventricular contractility changes. Anaesthetic agents have a similar effect on contractility. However, the fluid boluses were given after general anaesthesia had been induced, and the anaesthesia protocol was the same in all groups.

## Conclusions

This report points at issues that may confound conclusions based on a FC conducted under anaesthesia in surgery. The BV response to successive FCs was weaker for Ringer than for the colloid and the difference was even greater with respect to the SV response. The total ΔSV/ΔBV ratio for three consecutive FCs with of Ringer was only 10% of the colloid.

The suggested reasons for the differences include that pharmacokinetic properties of crystalloid fluid gradually attenuate and hence slow and reduce the BV expansion for repeated FCs, the Ringer BV expansion is proportionally less impactful on the stressed BV / preload. We speculate that Ringer’s fast extravascular distribution might impair the heart´s pumping capacity.

The poor SV response to a standard infusion volume of crystalloid fluid likely make interpretation of ΔSV% more difficult and is at risk of yield false negative determination of fluid non-responsiveness. Finally, there is an inflow of interstitial fluid to the plasma during 20 min after induction of general anaesthesia. This inflow has not previously been recognised to have a role in the interpretation of FCs, determination of Starling curve position and systemic DO_2_ levels.

## Author’s contributions

TOB: conceptualization, data curation, formal analysis, investigation, methodology, software, validation, writing - original draft, writing – review and editing.

RGH: conceptualization, data curation, formal analysis, software, supervision, validation, visualization, writing – review and editing.

## Consent for publication

Not applicable.

## Ethics approval and consent to participate

The study protocol was approved by the Ethics Committee of the First Affiliated Hospital, College of Medicine, Zhejiang University (Hangzhou, PR of China; No. 2011150, Official in charge: Zhangfei Shou) and registered at the Chinese Clinical Trial Registry (http://www.chictr.org/en; No. ChiCTR-TNRC-14004479). Written informed consent was obtained from each study subject.

## Funding

There was no specific funding for the present work.

## Availability of data and material

The original data is available as Supplementary File 2.xls

## Declaration of competing interest

RGH has received a grant from Grifols for studies of 20% albumin. TOB is CEO of TOB1 Consulting Ltd which has a patent application relating to fluid and hemodynamic monitoring.
