# Supplementary material for: Revisiting the fluid challenge and stroke volume optimisation after induction of general anaesthesia
Source: Ann Intensive Care. 2026 May 22;16:100093. doi: 10.1016/j.aicoj.2026.100093 (PMC13250664; doi:10.1016/j.aicoj.2026.100093)
Supplement: Supplementary file 1 [file mmc1.docx]

**Supplementary File 1 for**

**”**Revisiting the fluid challenge and stroke volume optimisation

after induction of general anaesthesia”

**__________________________________________________________________________**

***Calculation of Guyton´s parameters.***

An analogue to the mean circulatory filling pressure (P_msa_) has been derived from measurements of cardiac output, MAP, and CVP, assuming a constant veno-arterial compliance of 24:1 [16–18]

P_msa_ = *a* CVP + *b* MAP + *c* CO

where *a* = 0.96, *b* = 0.04 (*a* + *b* =1), and *c* = 0.96 x 1/26 x systemic vascular resistance at rest. However, *c* is commonly derived from anthropometric data. The value of *c* varies between 0.3 and 1.2 depending on age and body constitution (average 0.6) and is calculated as follows [16]:

*c* = 0.038 (94.17+0.193 age) / [4.5 (0.99 ^age-15^ 0.007184 (height^0.725^) weight^0.425^]

Pressure gradient for venous return (dVR) is obtained as: dVR = P_msa_ – CVP

The global pumping efficiency (Eh) is calculated as: Eh = (P_msa_ – CVP) / P_msa_

The resistance to venous return (RVR) was obtained as: RVR = dVR / CO

___________________________________________________________________________

***
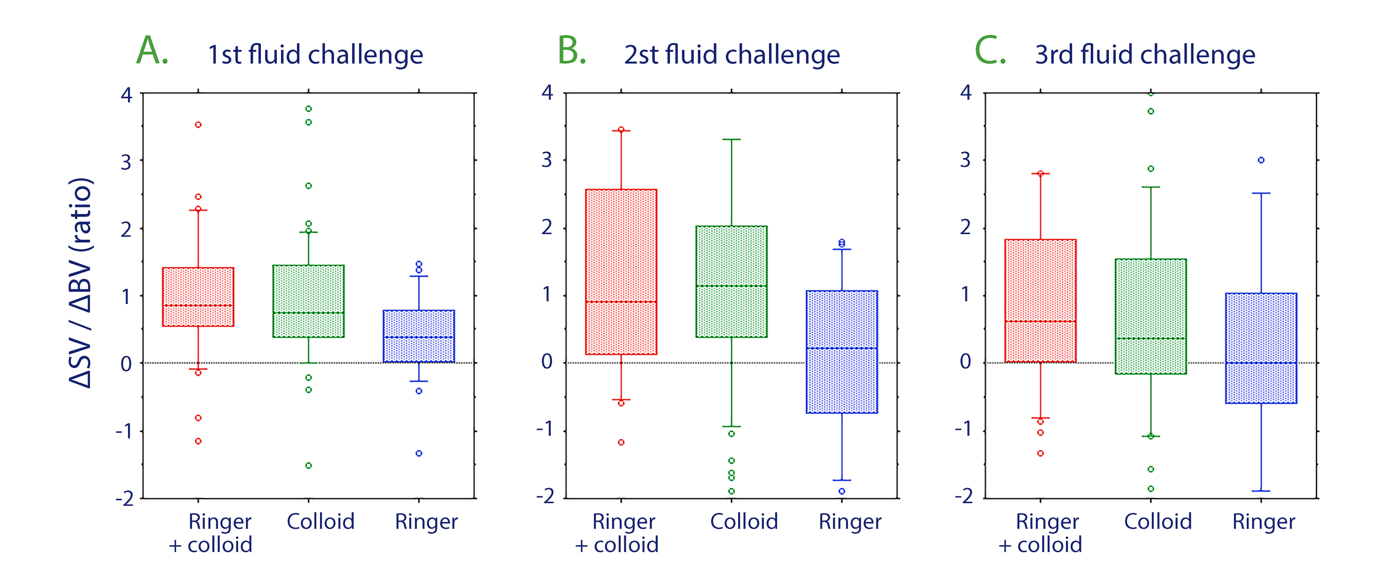
***

**Fig. S1.** Changes in the ratio between the relative changes in stroke volume and blood volume (∆SV%/∆BV%) at the end of 3 consecutive bolus infusions of 3 mL/kg of fluid according to 3 different programs.

**Fig. S2A. Haemodynamic measurements**

*Figures show median values for trends in the parameters displayed.*


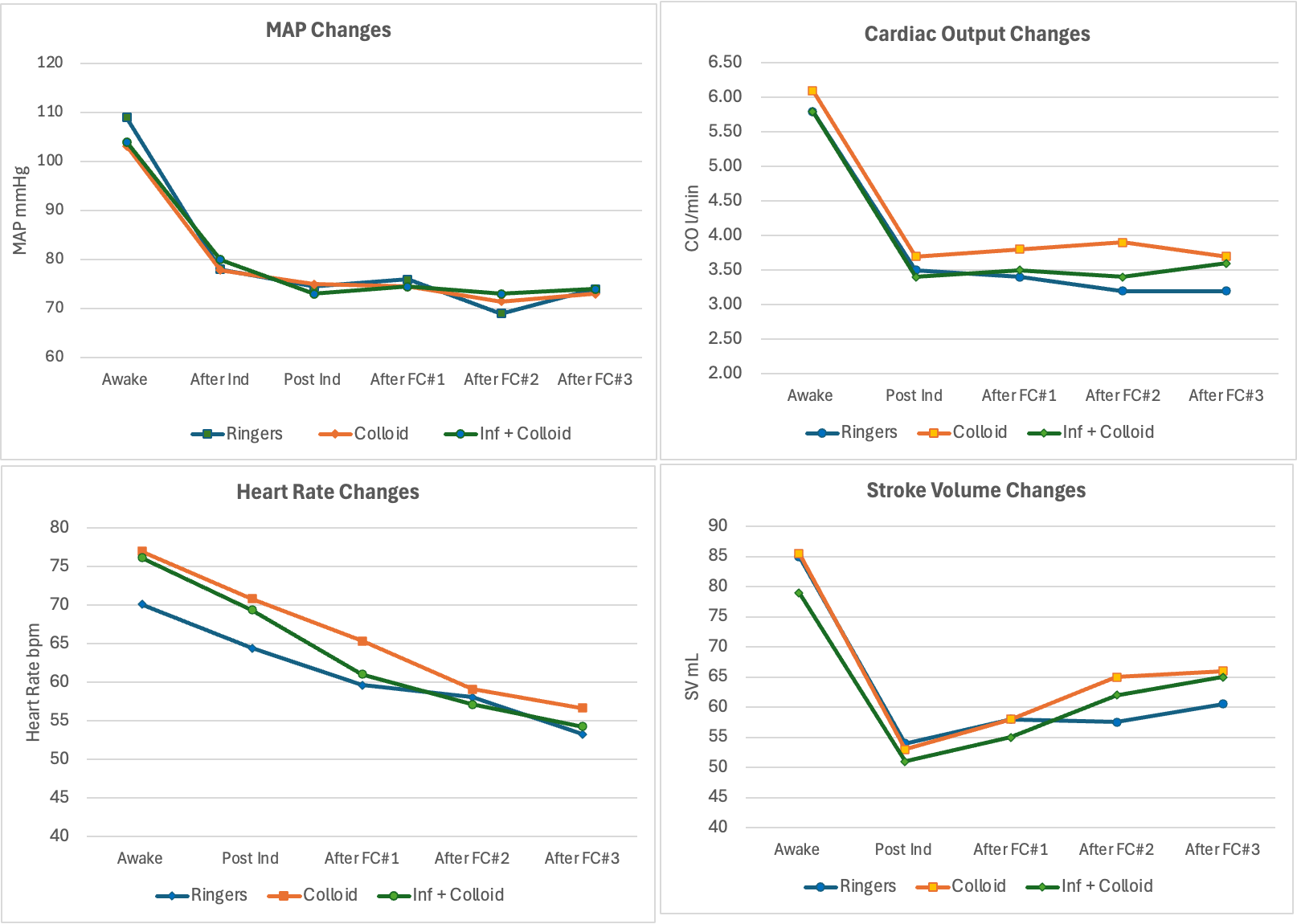


**Fig. S2B. Oxygen delivery (DO_2_)**


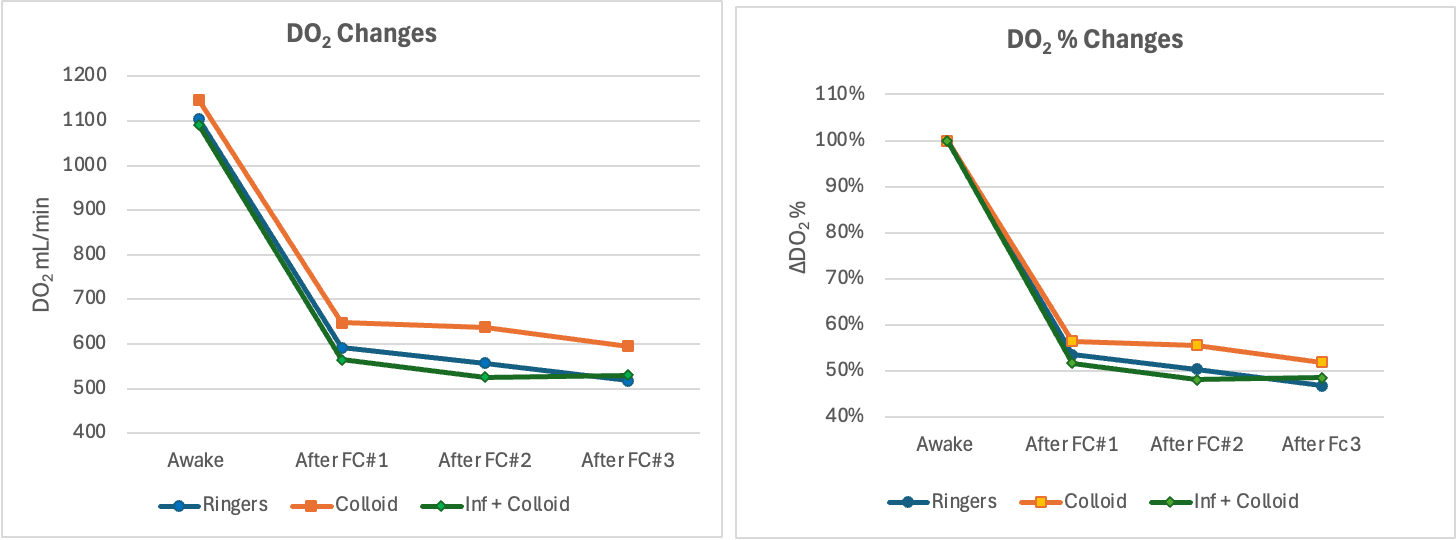


_________________________________________________________________________

**Fig. S3. Guyton´s haemodynamic parameters**

*Figures show median values for trends in the parameters displayed.*


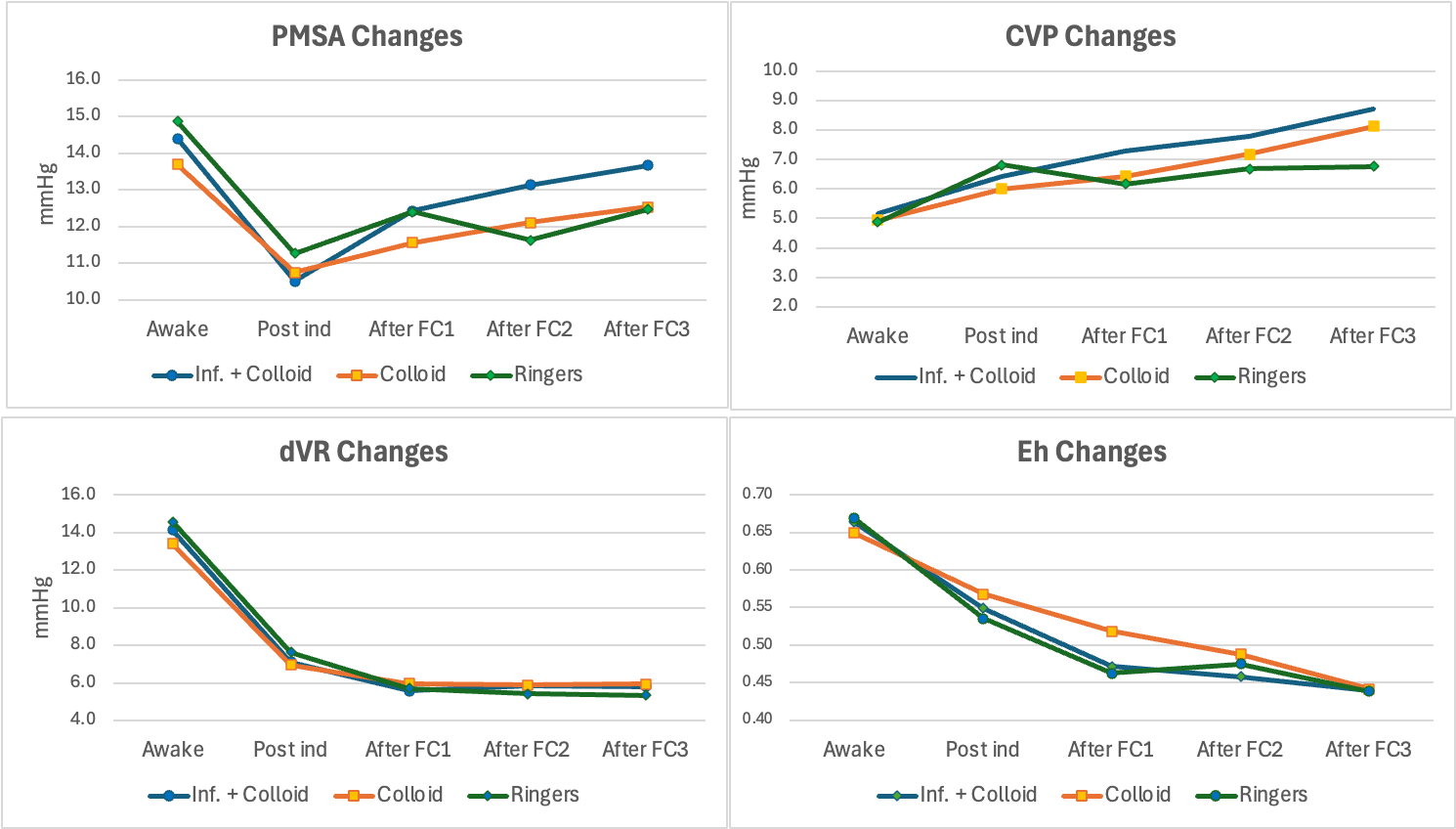


_________________________________________________________________________
